# Intrinsic Organization of Occipital Hubs Predicts Depression: A Resting-State fNIRS Study

**DOI:** 10.3390/brainsci12111562

**Published:** 2022-11-17

**Authors:** You Xu, Yajie Wang, Nannan Hu, Lili Yang, Zhenghe Yu, Li Han, Qianqian Xu, Jingjing Zhou, Ji Chen, Hongjing Mao, Yafeng Pan

**Affiliations:** 1 Department of Sleep Medicine, Affiliated Mental Health Center & Hangzhou Seventh People’s Hospital, Zhejiang University School of Medicine, Tianmushan Road 305, Hangzhou 310013, China; 2 Department of Psychology and Behavioral Sciences, Zhejiang University, Hangzhou 310058, China; 3 Department of Psychiatry, Affiliated Mental Health Center & Hangzhou Seventh People’s Hospital, Zhejiang University School of Medicine, Hangzhou 310013, China

**Keywords:** depression, cortical networks, fNIRS, resting state, connectome

## Abstract

Dysfunctional brain networks have been found in patients with major depressive disorder (MDD). In this study, to verify this in a more straightforward way, we investigated the intrinsic organization of brain networks in MDD by leveraging the resting-state functional near-infrared spectroscopy (rs-fNIRS). Thirty-four MDD patients (24 females, 38.41 ± 13.14 years old) and thirty healthy controls (22 females, 34.43 ± 5.03 years old) underwent a 10 min rest while their brain activity was recorded via fNIRS. The results showed that MDD patients and healthy controls exhibited similar resting-state functional connectivity. Moreover, the depression group showed lower small-world Lambda (1.12 ± 0.04 vs. 1.16 ± 0.10, *p* = 0.04) but higher global efficiency (0.51 ± 0.03 vs. 0.48 ± 0.05, *p* = 0.03) than the control group. Importantly, MDD patients, as opposed to healthy controls, showed a significantly lower nodal local efficiency at the left middle occipital gyrus (0.56 ± 0.36 vs. 0.81 ± 0.20, p_FDR_ < 0.05), which predicted the level of depression in MDD (*r* = 0.45, *p* = 0.01, *R*^2^ = 0.15). In sum, we found a more integrated brain network in MDD patients with a lower nodal local efficiency at the occipital hub, which could predict depressive symptoms.

## 1. Introduction

Major depressive disorder (MDD) is a disabling disease associated with profound functional impairment [1]. According to the World Health Organization, before the COVID-19 pandemic, approximately 350 million people worldwide were suffering from depression [2]. Estimates put the rise in both anxiety and depressive disorders at more than 25% during the first year of the pandemic [3]. In addition to the core emotional symptom, cognitive subdomains, such as learning and memory, executive functioning, processing speed, and attention and concentration, are significantly impaired in patients with MDD [4]. However, due to the lack of specific diagnostic methods for depression and its various manifestations, accurate and reliable diagnosis of MDD mainly depended on the subjective assessment and clinical experience of the clinicians. Thus, the accuracy of reaching a diagnosis, relying on past experience remains debated [5]. Biomarkers offer a conceivable target for assisting in diagnosis and identifying predictors of response to various interventions [6]. Brain imaging techniques are powerful tools for tracking biomarkers, and thus have been used as an adjunct to help clinicians diagnose MDD in recent years.

Functional near-infrared spectroscopy (fNIRS) is a non-invasive, functional neuroimaging technique that could assess cerebral function by quantifying cerebral oxygenation [7,8,9,10]. This type of spectroscopy, fNIRS, penetrates organic tissues by using light sources with a spectral window of 650 to 1000 nm. The variance in absorbance is then used to compute variations in oxygenated hemoglobin (HbO) using the modified Beer–Lambert law. This noninvasive method can measure the hemoglobin’s level of cortical oxygenation [5] This technology has extended to the clinical field of psychiatry as one of the objective modalities for probing psychoses [11]. Its applicability, safety, low cost, ecological validity, tolerance to movements, and non-involvement of nonionizing radiation make fNIRS advantageous for scientific research and clinical applications [8]. Some researchers suggested that some features in fNIRS could serve as candidate biological markers for aiding the diagnosis of psychosis spectrum in routine settings [12,13,14]. The similarity in cortical activity in schizophrenia and bipolar disorder, compared to healthy controls pointed to a possible neurobiological convergence of schizophrenia and bipolar disorder in underlying impairments of social cognition [15]. Meanwhile, a combined index of two task-evoked features in HbO changes during a VFT could differentiate individuals with psychiatric disorders or mood disorders from healthy controls [16]. Patients with affective disorders, including major depressive disorder and bipolar disorder, exhibited significantly reduced intra-regional and symmetrically interhemispheric connectivities in the prefrontal cortex when compared to healthy controls [17]. Some researchers found lower activity in individuals with bipolar disorder in the depression phase during cognitive tasks. They suggested the single-trial symbol check task to be helpful for the diagnosis of bipolar depression [18]. Disrupted prefrontal cortex activity was found in patients with bipolar disorder and borderline personality disorder and is more extensive in borderline personality disorder [19].

As a common psychosis, depression also presents abnormalities on brain activity, as measured by fNIRS. The application of fNIRS consistently demonstrated attenuated cerebral hemodynamic changes in those with depression, compared to healthy individuals when utilizing the verbal fluency task (VFT) as the active paradigm [5,20]. The fNIRS measurement of the cerebral cortex under emotional- or cognitive-related tasks is suggested to serve as a supplementary test to support the diagnosis of MDD [21,22]. The task-based decrease in oxygenated hemoglobin (HbO) concentration was related to the severity of depression [23,24,25]. In addition, the distinct pattern of activation of the cortex on regional changes in oxy-Hb may reflect specific functional abnormalities within different subtypes of depression [26], and it may help to distinguish MDD from other mental disorders, such as bipolar disorder [27,28], generalized anxiety disorder (GAD) [29], and borderline personality disorder [30]. The majority of the fNIRS studies adopted protocols of tasks to distinguish the depressed from the healthy [5]. However, task-based studies may be impacted by confounds, such as levels of motivation, fatigue, and disinterest [31].

The non-task (i.e., doing nothing) is a resting state that is easy to implement. The spontaneous brain activity during the non-task period can be used as a baseline reference for brain activation [32,33], which was associated with a variety of neuropsychiatric disorders [34]. When considering the magnitude spectrum and average power of the cerebral hemoglobin fluctuation, the non-task may not be sufficient to separate major depression or combined anxiety and depression from healthy controls [35]. Resting-state functional connectivity (RSFC), an inherent feature of the brain that consists of slow spontaneous oscillations during rest or sleep, has been utilized to study neurological disorders [17]. RSFC in the default mode network (DMN) was decreased in depressed subjects, compared to non-depressed subjects—an effect that is partly associated with the process of mind-wandering and state/trait rumination [36]. Notwithstanding, the intrinsic organization of RSFC based on resting-state fNIRS (rs-fNIRS) in MDD remains incompletely investigated. Furthermore, relationships between networks are altered in depression, in addition to the connectivity patterns within the core resting-state connectivity networks [37,38]. The disrupted topological architecture of functional brain networks was observed in MDD using resting-state functional MRI (rs-fMRI) [39]. Whether this abnormity could be detected by rs-fNIRS in a more straightforward way was not clear.

### Hypotheses

The first hypothesis is that the dysfunction of the brain network in patients with MDD can be detected by rs-fNIRS. Thus, we sought to perform an rs-fNIRS based functional connectivity analysis to investigate the intrinsic organization of brain networks in MDD. Moreover, we have the second hypothesis that the disrupted topological architecture of the brain network can be found in patients with MDD using rs-fNIRS, which could help distinguish patients from healthy people. Therefore, we calculated graph theory-based connectivity metrics to quantitatively characterize functional connectivity in each group, expecting to find some brain indices that helps differ MDD to healthy individuals.

## 2. Methods

### 2.1. Participants

A total of 64 adults, including 34 participants (24 females, mean age ± SD = 38.41 ± 13.14 years old) in the depression group and 30 participants (22 females, 34.43 ± 5.03 years old) in the healthy control group, participated in the current study. All patients were in-patients who volunteered to take part in this study and provided their consent forms, and their admission dates ranged from 25 February 2021 to 31 August 2021. The patients with depression were recruited from the Hangzhou Seventh People’s Hospital, while the healthy controls were recruited through advertisements. All the patients with depression met the American Psychiatric Association DSM-IV diagnostic criteria of depression, and healthy controls were interviewed using the Structured Clinical Interview for DSM-IV, nonpatient edition. Age and sex were matched between the two groups (age: *t* = 1.56, *p* = 0.12; sex: *χ*^2^ = 2.08, *p* = 0.20). In the non-depressive sample, 6.67% (*n* = 2) of people had a doctorate degree, 23.33% (*n* = 7) had a master’s degree, 63.33% (*n* = 19) had a university degree, and 6.67% (*n* = 2) had an associate degree. A total of 17.65% (*n* = 6) of the depressed sample has a university degree, 11.76% (*n* = 4) had an associate degree, 20.59% (*n* = 7) had a high school diploma, and 50% (*n* = 17) had a middle school degree. A total of 61.76% (*n* = 21) of the clinical sample had a generalized anxiety disorder; 14.71% (*n* = 5) of all depressive patients were diagnosed with non-organic sleep disorders and other comorbidities, such as sleep apnea syndrome (5.88%, *n* = 2), obsession (5.88%, *n* = 2) and panic attacks (5.88%, *n* = 2). Hamilton Depression Rating Scale (HAMD) [40] was used to assess the depression level in both groups (depression vs. control, 22.55 ± 1.27 vs. 6.66 ± 1.51, *t* = 17.09, *p* < 0.001, Cohen’s *d* = 11.39). The study procedure was carried out following the Declaration of Helsinki and approved by the ethics committee of local hospital (No. 2021024). All participants gave their written informed consent before the experiment. 

### 2.2. NIRS Data Acquisition

**Figure 1:**
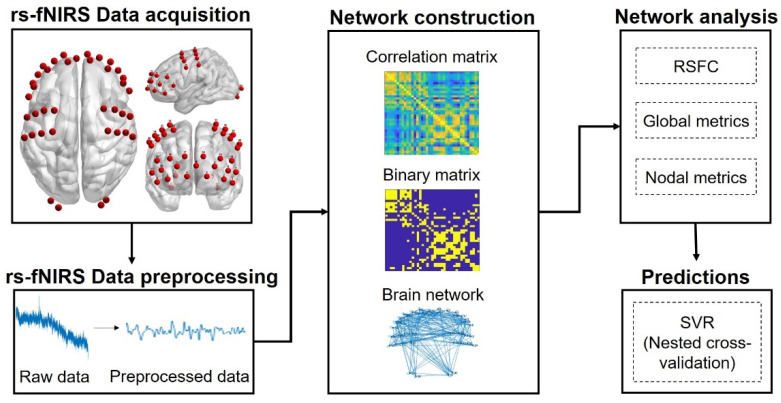
Overview of resting-state functional near-infrared spectroscopy (rs-fNIRS) channel locations and analytical pipeline. RSFC, resting-state functional connectivity. SVR, support vector regression.

We collected our resting-state data using a multi-channel functional near-infrared spectroscopy (fNIRS) optimal imaging system (NirScan-900A, Huichuang, China), equipped with 15 detectors and 19 sources, resulting in 39 measurement channels in total to cover prefrontal, central, and posterior cortices (see Figure 1 for the locations of measurement channels). The distance of source-detector separation was approximately 3 cm. The absorption of near-infrared light at three wavelengths (730, 808, and 850 nm) was recorded with a sampling rate of 50 Hz. The light absorption data were converted into concentrations of oxy-hemoglobin (HbO) and deoxy-hemoglobin (HbR) using a modified Beer–Lambert law [41], with a differential pathlength factor of 6 [42]. We focused on HbO signals given the higher signal-to-noise than HbR [43]. For each participant, the rs-fNIRS data were collected for 10 min, during which the participants were instructed to keep relaxed, keep their eyes open, and remain awake. 

### 2.3. NIRS Data Preprocessing

We used the Homer 2 toolbox and custom codes in MATLAB 2022a to preprocess rs-fNIRS data [44]. Standardized preprocessing entailed the following steps. First, bad channels were identified (i.e., default values in HoMER2: optical density < 0 or > 1 × 10^7^, or signal-to-noise < 2) and pruned (function enPruneChannels). Next, motion artifacts were identified and corrected by a cubic spline interpolation method (function hmrMotionArtifactByChannel) with input parameters: tMotion = 0.5, tMask = 1, STDEVthresh = 30, AMPthresh = 0.5 [45]. The data were band-pass filtered (0.01–0.08 Hz) to extract spontaneous neural activity. A wavelet-based denoising method was used to remove superficial physiological noise and its related spurious connectivity [46]. Preprocessed signals were entered into subsequent analyses. 

### 2.4. Network Construction

Network construction encompassed two steps. First, in accordance with previous studies [47], Pearson correlation coefficients were used to quantify the relationships among time courses of every pair of channels, resulting in a 39 × 39 correlation matrix. We zeroed all the negative correlation coefficients because of their ambiguous biological meanings [48,49]. We restricted following analyses to only positive correlations. All correlation coefficients were subjected to Fisher’s *z*-transformation to improve normality.

Second, we binarized the correlation matrix according to sparsity-based threshold [50,51]. The correlation matrix was thresholded over a range of sparsity (from 20% to 40%, with 1% step size) in order to investigate the relationship between sparsity and the network properties. Specifically, each functional connectivity matrix *Z*_ij_ can be converted to a binarized matrix *B*_ij_, where *B*_ij_ is 1 if the value of the *z* value in matrix *Z*_ij_ is greater than a given sparsity threshold and 0 otherwise [52]. Consistent with recent recommendations [52], we also adopted a single sparsity (25%) to normalize the network metrices, thereby allowing the comparison of group differences under the same topological organizations.

### 2.5. Network Analysis

In the current study, we used the GRETNA toolbox [53] in MATLAB 2022a to assess the topological measures, including global network metrics (small-world Gamma, Lambda, Sigma, global efficiency, and local efficiency) and regional nodal metrics (global nodal efficiency and local nodal efficiency) for the brain network.

#### 2.5.1. Global Network Metrics

*Small world*. We focused on two key properties of the small world in a graph *G*: the cluster coefficient (*C_p_*) and the characteristic path length (*L_p_*) [54]. *C_p_* is the average of cluster coefficients over all nodes in the network. One-node cluster coefficient refers to the number of existing edges between that node and its neighbors, divided by the number of all theoretically possible edges [55]. For a given graph *G* with *n* nodes and *K* edges, the *C_p_* of the graph *G* is computed as follows [54]:

$$C_{p}=\frac{1}{N}\underset{i\in G}{\sum }\frac{E_{i}}{D_{i}\left(D_{i}-1\right)/2}$$

where *D_i_* is the number of edges connected to node *i*. *E_i_* denotes the number of edges in the subgraph. *C_p_* reflects the local interconnectivity and cliquishness of a brain network [47]. 

The *L_p_* refers to the average of the shortest path lengths between all pairs of nodes in the network, whereas the shortest path length is defined as the minimum edges that link arbitrary nodes [55]. The *L_p_* of a graph *G* is defined as the average of the shortest path lengths between all pairs of nodes in network [52,54]:

$$L_{p}=\frac{1}{N\left(N-1\right)}\underset{i\neq j\in G}{\sum }d_{ij}$$

where *d_ij_* represents the shortest path length between node *i* and node *j*. Therefore, *L_p_* reflects the ability of serial information propagation within the network [55]. 

To examine the small-world attributes of a network, the normalized *C_p_* (referred to as *Gamma*) and normalized *L_p_* (referred to as *Lambda*) were calculated. Gamma and Lambda were computed as the ratio of the values to random rewired networks [56]:

$$\mathrm{Gamma}=\frac{C_{p}^{real}}{C_{p}^{rand}},\;\mathrm{Lambda}=\frac{L_{p}^{real}}{L_{p}^{rand}}$$

$C_{p}^{real}$ and $L_{p}^{real}$ denote the clustering coefficient and the characteristic path length of a real network, whereas $C_{p}^{rand}$ and $L_{p}^{rand}$ are the means of the same parameters derived from 1000 matched random networks. The random network has the same number of nodes and edges and the same distribution of degrees as the real one. The ratio of Gamma to Lambda is defined as *Sigma* [57]:

$$\mathrm{Sigma}=\frac{\mathrm{Gamma}}{\mathrm{Lambda}}$$

A small-world network is typically characterized as Lambda ≈ 1, Gamma > 1, and Sigma > 1 [58].

*Efficiency*. We also computed two properties of network regarding its efficiency, i.e., global efficiency and local efficiency. Global efficiency is defined as the inverse of the harmonic mean of the shortest path lengths between two arbitrary nodes in the entire network [55]. Global efficiency is calculated as follows:

$$E_{glob}=\frac{1}{N\left(N-1\right)}\underset{i\neq j\in G}{\sum }\frac{1}{d_{ij}}$$

where *d_ij_* represents the shortest path length between node *i* and node *j*. Global efficiency reflects parallel information transformation at the global level in a network [50].

Local efficiency is computed as the average efficiencies of all nodes [55], wherein one given node and its direct neighbors compromised a sub-network. Local efficiency is calculated as follows:

$$E_{loc}=\frac{1}{N}\underset{i\in G}{\sum }E_{glob}\left(i\right)$$

where *E_glob_* is the global efficiency of *G_i_*, which is the subgraph of the neighbors of node *i*. According to its definition, local efficiency can be regarded as a measure of information transfer within the immediate neighborhood of each node [50].

#### 2.5.2. Regional Nodal Metrics

Complementary to global network metrics, we assessed *nodal global efficiency* and *nodal local efficiency* to provide measures for efficiency at regional level. For an indexed node, nodal global efficiency (also known as nodal efficiency) is defined as the inverse of the harmonic mean of minimum path length between that node and all other nodes in the network [50,59]:

$$E_{nod}\left(i\right)=\frac{1}{N-1}\;\underset{j\neq i\in G}{\sum }\frac{1}{d\left(i,j\right)}$$

where *d*(*i,j*) denotes the shortest path length between node *i* and node *j*. It measures how well a sub-group is integrated in the whole network and reflects the ability of information exchange of the node itself [60]. 

The nodal local efficiency is measured as follows:

$$E_{loc}\left(i\right)=\frac{1}{N_{G_{i}}\left(N_{G_{i}}-1\right)}\underset{j\neq i\in G_{i}}{\sum }\frac{1}{d\left(i,j\right)}$$

where *G_i_* is the subgraph that includes node *i* and all its direct neighbors. Nodal local efficiency measures the communication ability of a sub-network consisting of the node itself and its direct neighbors [60]. 

The node of high efficiency is important for information integration and distribution [61]; in this study, the nodes with higher values in nodal efficiency (at least 1 SD larger than the average of all nodes in the brain network) were defined as brain hubs that are typically assumed to play critical roles in the functional integrity of whole networks [52]. BrainNet Viewer was used for visualization of regional nodal properties [62]. Channel-wise independent-sample *t*-tests were conducted to compare the group differences in nodal global efficiency and nodal local efficiency, with the false-discovery-rate (FDR) method accounting for the multiple comparisons [63].

### 2.6. Support Vector Regression

To explore whether it is possible to predict depression level (as assessed by HAMD scores) based on brain network metrices, we used the epsilon-support vector regression (*ε*-SVR, [64]). The radial basis function was utilized to construct the non-linear SVR model. We employed a grid search-based approach for hyperparameter optimization to determine the optimal regression parameters (i.e., *C*, *γ*, and *ε*). A nested cross-validation method was implemented [65], with the outer leave-one-out cross validation (LOOCV) estimating the generalization performance of the model and inner 10-fold CV estimating and selecting the optimal hyperparameters. The prediction accuracy was estimated using the Pearson correlation coefficient between the predicted and actual values [66]. The coefficient of determination (denoted by *R*^2^) was also reported. We used the *libsvm* toolbox and custom codes in MATLAB to perform SVR analyses [67]. 

## 3. Results

### 3.1. Resting-State Functional Connectivity

**Figure 2:**
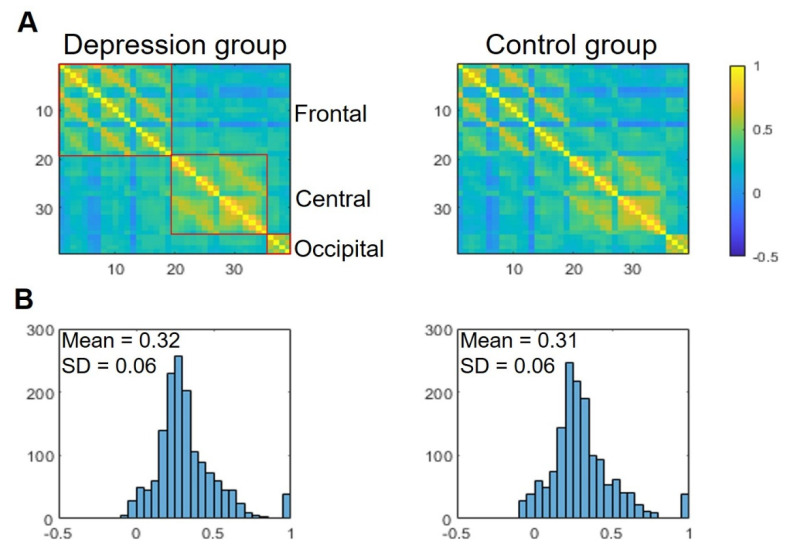
(**A**) The averaged group-level resting-state functional connectivity (as indexed by *r* value correlation matrices) of the depression and control groups. Digits in matrices indicate measurement channels. Frontal, central, and occipital channels are clustered and highlighted. (**B**) The distribution of correlation matrices of the two groups.

The RSFC pattern at the group level for patients with depression and healthy controls were illustrated in Figure 2. To explore whether there were significant differences in RSFC between the two groups, a series of independent-sample *t*-tests were conducted. Interestingly, the results showed that patients with depression (M ± SD, 0.32 ± 0.06, Pearson correlation coefficients) and healthy controls (0.31 ± 0.06) exhibited similar RSFC. Thus, RSFC analysis was insufficient to discriminate between the two groups in this study. 

### 3.2. Global Network Properties

**Figure 3:**
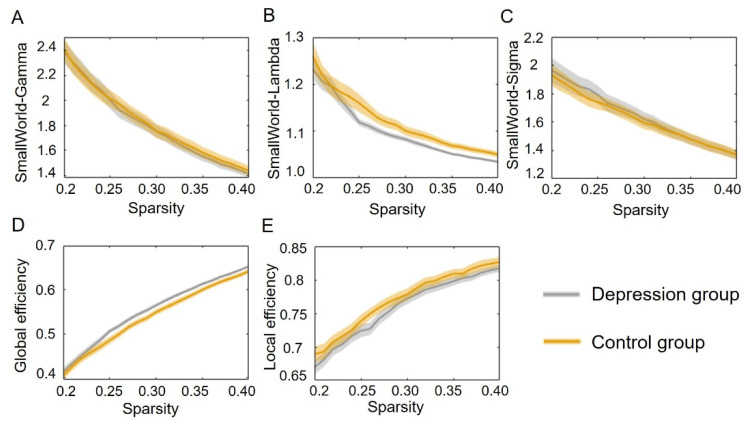
The global network metrices in a range of sparsity thresholds (20–40%). Panels (**A**–**E**) refer to small-world Gamma, small-world Lambda, small-world Sigma, global efficiency, and local efficiency as functions of sparsity thresholds. The shadows indicate standard error in all participants.

To further investigate the intrinsic organization of cortical networks, we next analyzed global network properties, including small-world parameters (Gamma, Lambda, and Sigma) and global and local efficiencies, for the two groups. Figure 3 shows the profiles of five global network properties as functions of the sparsity thresholds (ranging from 20% to 40%, with 1% step size). We observed that the depression group showed lower small-world Lambda but higher global efficiency than the control group. 

**Figure 4:**
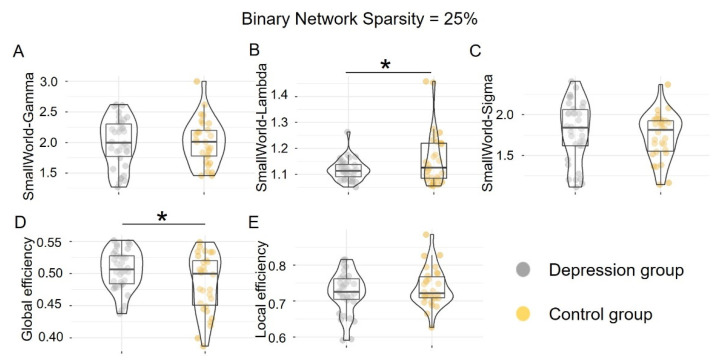
Group differences in five global properties in binary brain networks (sparsity threshold = 25%). The upper three panels (**A**–**C**) correspond to the small-world metrics: Gamma, Lambda, and Sigma, respectively. The lower two panels (**D**,**E**) refer to global and local efficiency. * *p* < 0.05.

In accordance with recent studies [52], we adopted a single sparsity (i.e., 25%) to normalize all of the networks to explore the group differences in the same-size network topological organization. We measured lower Lambda in the depression group (1.12 ± 0.04), as relative to the control group (1.16 ± 0.10, *t* = 2.11, *p* = 0.04, Cohen’s *d* = 0.53). We also found that the depression group (0.51 ± 0.03) showed higher global efficiency, compared to the control group (0.48 ± 0.05, *t* = 2.19, *p* = 0.03, Cohen’s *d* = 0.73; Figure 4). 

### 3.3. Regional Nodal Properties

**Figure 5:**
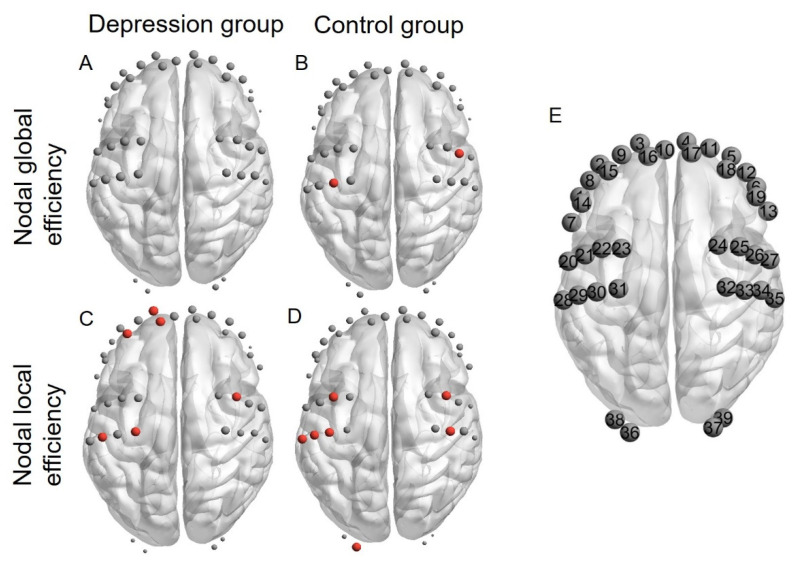
Regional nodal properties and hub distributions in the depression and control groups. Each dot means a single channel, and red dots represent hubs (see Methods). Panels (**A**,**B**) depict nodal global efficiency in the depression and control groups, respectively; panels (**C**,**D**) denote the nodal local efficiency in the depression and control groups, respectively. (**E**) Numbered channels for clarity.

Having established the group differences in global network properties, we then tested the differences between the depression and control groups in terms of their regional nodal properties. Regarding nodal global efficiency, we found no hub in the depression group, but detected two central hubs (channels 26 and 30) in the control group (Figure 5A,B). Concerning nodal local efficiency, we found three frontal hubs (channels 3, 15, and 16) and three central hubs (channels 25, 31, and 39) in the depression group and six central hubs (channels 22, 25, 28, 29, 30, and 33) and one occipital hub (channel 36) in the control group (Figure 5C,D). Critically, the depression group (0.56 ± 0.36) compared to the control group (0.81 ± 0.20) showed a significantly lower nodal local efficiency at channel 36 (*t* = 3.38, *p_FDR_* < 0.05, Cohen’s *d* = 0.86), which roughly corresponds to the left middle occipital gyrus [68]. As a robustness check, we additionally ran a one-way ANCOVA on the nodal local efficiency at channel 36, with HAMD scores and medication usage as covariates. The results showed that the group effect was still significant (*F* = 10.87, *p* = 0.0017, η_p_^2^ = 0.16). There were no significant effects for the covariates (*F* = 0.98, *p* = 0.37 for HAMD score and *F* = 0.24, *p* = 0.62 for medication usage). No significant group differences in nodal global efficiency were found (all *p_FDR_* > 0.05).

### 3.4. Prediction of the Depression Level

**Figure 6:**
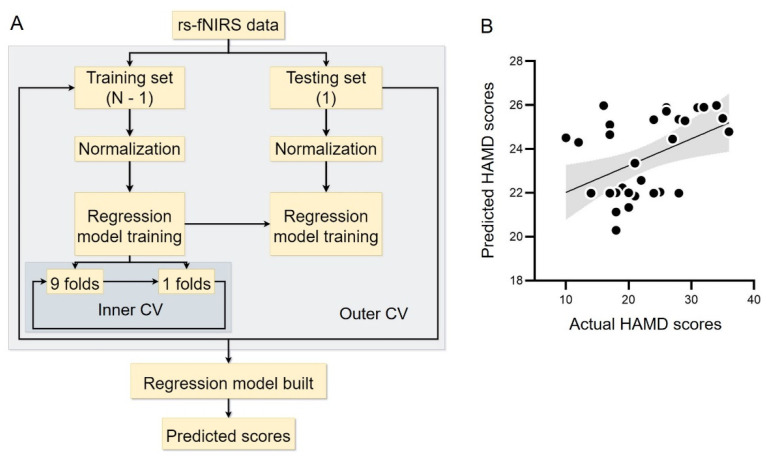
(**A**) The flow chart of prediction analyses based on the support vector regression (SVR) model. (**B**) The correlation between predicted and actual HAMD scores in the depression group. The shadow indicates 95% confidence interval. Rs-fNIRS, resting-state functional near-infrared spectroscopy. CV, cross validation. HAMD, Hamilton Depression Rating Scale.

Finally, we sought to test whether it is possible to predict the depression level based on intrinsic organization of cortical networks in the depression group. To this end, we constructed *ε*-SVR models with nested cross-validation (Figure 6A). We extracted global and local network properties, respectively, for each participant as predictors. The depression level, as measured by the HAMD scores, was the outcome variable. A data-driven method was used to search for the best predictor for the prediction analyses (one type of network properties as the predictor each time). We found that only when the occipital hub (i.e., channel 36) was derived from the nodal local efficiency and used as a predictor, was the correlation between actual and predicted HAMD scores significant (*r* = 0.45, *p* = 0.01, *R*^2^ = 0.15) in the depression group (Figure 6). These results indicate that it is possible to infer the depression level based on patients’ nodal local efficiency in the occipital hub. 

## 4. Discussion

In the present study, we performed functional connectivity-based comparisons between patients with depression and healthy controls. The results showed that patients with depression and healthy controls exhibited similar RSFC. However, the depression group showed lower small-world Lambda and higher global efficiency than the control group. The depression group compared to the control group showed a significantly lower nodal local efficiency at the occipital hub (i.e., channel 36), which roughly corresponds to the left middle occipital gyrus. When the occipital hub (channel 36) was derived from the nodal local efficiency and used as a predictor, the correlation between actual and predicted HAMD scores was significant in the depression group.

Resting-state functional MRI (rs-fMRI) revealed reduced nucleus accumbens functional connectivity in default mode network (DMN) in patients with recurrent major depressive disorder [69]. However, the decreased DMN functional connectivity was related with medication usage but not with MDD duration, as it was not found in first-episode drug-native MDD [70]. In this study, our results did not support our first hypothesis. The MDD patients were not drug-naïve, and it is possible that the drug usage in our patients might explain why their RSFC is similar to the healthy controls.

Our second hypothesis was verified. In this study, a more integrated brain network (lower Lambda and higher global efficiency) was found in MDD patients despite some negative results (Gamma, Sigma, and local efficiency). A review using non-invasive neuroimaging data and graph theoretical approaches for psychiatric disorders found that patients with depression did not display consistent alterations in small-world properties [71]. However, a significant increase in Gamma and Sigma despite the increase in Lambda was found in patients with MDD after electroconvulsive therapy [72]. It suggested that the Lambda may be a more stable trait biomarker of depression, despite the treatment. Some researchers detected a decreased global efficiency within MDD patients [39,73,74], which may be related to negative affective processing [75]. Other studies found no significant differences between MDD and healthy controls in terms of global efficiency [76,77], or generally higher global efficiency in MDD individuals [78]. Increased local efficiency, which associated with high HAMD score, was found in individuals with depression symptoms [79,80]. That indicated excessively high network segregation which might increase the brain’s overall wiring cost [81]. Aforementioned studies were based on fMRI or EEG, while the present study was based on fNIRS, which found a different topological change in the brain network of MDD patients. It is likely that the metric of global efficiency might be sensitive to subtypes of depression, which deserves future investigations.

Crucially, this study found the MDD patients had significantly lower nodal local efficiency at the occipital gyrus that could predict HAMD scores. Our results echo a previous study that indicated aberrant nodal efficiency and centrality of regional connectivity in the occipital cortex [82]. Regional homogeneity (ReHo) was found to be lower in the occipital gyrus among MDD patients, which could even help to discriminate patients with melancholic MDD from patients with non-melancholic MDD [83]. In addition, MDD patients had abnormal local intrinsic gray-matter connectivity in the occipital cortex, which was associated with some symptoms of depression [84]. These results might help explain the aberrant topological properties of brain functional connectivity at the occipital hub we found in MDD patients. Researchers found a smaller gray matter volume in the left occipital middle gyrus in MDD patients, compared with controls [85,86]. As for white matter, fractional anisotropy in the left middle occipital gyrus was reduced in MDD patients [87]. Meanwhile, MDD patients had decreased cerebral blood flow in the left middle occipital gyrus, especially in those with acute phase and medication-free [88]. These results might be a structural basis of the lower nodal local efficiency at the left middle occipital gyrus. However, the abnormal activity in the left middle occipital gyrus may be state-specific in current and remitted MDD patients [89]. That may help explain some inconsistent results [90].

Our findings at the occipital hub could also be interpreted from an “information processing” perspective [91]. A recent study found that the amplitude of low-frequency fluctuations in the left middle occipital gyrus decreased within patients with MDD, compared to healthy controls; the researchers argued that the left middle occipital gyrus may be involved in the processing of cognitive biases of MDD in resting states [92]. Consistent with this research, decreased nodal local efficiency at the middle occipital gyrus bolstered the notion that processing bias in MDD may be initiated as a perceptual visual bias. Biased information from the occipital hub could then spread through the brain due to the higher global efficiency in MDD patients. As the DMN hyperactivity was related to negative rumination in depression [93], the biased information initiated from the occipital hub may, eventually, cause a series of cognitive and affective symptoms of MDD. 

### Limitations and Strengths

There were some limitations in our study. Firstly, the MDD patients were not medication-naïve in this study. Thus, we could not exclude the possibility that these drugs affected the current results. Secondly, the sample was inadequate in looking for a differential effect across depressive subtypes. Thirdly, fNIRS has a penetration limit into the superficial gray matter of the cortex of around 2 cm. Therefore, we could not detect subcortical changes. Nevertheless, the topology of the cortex in MDD patients did show some abnormities. The main strength of this study is that we used a more convenient way of rs-fNIRS combined with graph theory to distinguish patients with MDD from healthy people.

## 5. Conclusions

In conclusion, we found a more integrated brain network with lower Lambda and higher global efficiency in MDD patients. Meanwhile, MDD patients had a lower nodal local efficiency at the occipital hub, which could predict depressive symptoms. These results may provide a new method in assisting the clinical diagnosis and help to elucidate the brain mechanism of MDD. Future studies could verify these results in subgroups of MDD patients, such as untreated patients, patients in remission stage, male and female patients, young and old patients, and so on. In addition, the other regions or areas should be explored.

## Data Availability

The data presented in this study are available on request from the corresponding author. The data are not publicly available due to restrictions of privacy and ethic.

## References

[1] Atique-Ur-Rehman H., Neill J.C. (2019). Cognitive Dysfunction in Major Depression: From Assessment to Novel Therapies. Pharmacol. Ther..

[2] James S.L., Abate D., Abate K.H., Abay S.M., Abbafati C., Abbasi N., Abbastabar H., Abd-Allah F., Abdela J., Abdelalim A. (2018). Global, Regional, and National Incidence, Prevalence, and Years Lived with Disability for 354 Diseases and Injuries for 195 Countries and Territories, 1990–2017: A Systematic Analysis for the Global Burden of Disease Study 2017. Lancet.

[3] WHO Make Mental Health & Well-Being for All a Global Priority. https://www.who.int/news-room/events/detail/2022/10/10/default-calendar/world-mental-health-day-2022---make-mental-health-and-well-being-for-all-a-global-priority.

[4] Pan Z., Park C., Brietzke E., Zuckerman H., Rong C., Mansur R.B., Fus D., Subramaniapillai M., Lee Y., McIntyre R.S. (2019). Cognitive Impairment in Major Depressive Disorder. CNS Spectr..

[5] Ho C.S.H., Lim L.J.H., Lim A.Q., Chan N.H.C., Tan R.S., Lee S.H., Ho R.C.M. (2020). Diagnostic and Predictive Applications of Functional Near-Infrared Spectroscopy for Major Depressive Disorder: A Systematic Review. Front. Psychiatry.

[6] Strawbridge R., Young A.H., Cleare A.J. (2017). Biomarkers for Depression: Recent Insights, Current Challenges and Future Prospects. Neuropsychiatr. Dis. Treat..

[7] Bahrani A.A., Kong W., Shang Y., Huang C., Smith C.D., Powell D.K., Jiang Y., Rayapati A.O., Jicha G.A., Yu G. (2020). Diffuse Optical Assessment of Cerebral-autoregulation in Older Adults Stratified by Cerebrovascular Risk. J. Biophotonics.

[8] Pan Y., Borragán G., Peigneux P. (2019). Applications of Functional Near-Infrared Spectroscopy in Fatigue, Sleep Deprivation, and Social Cognition.

[9] Pan B., Huang C., Fang X., Huang X., Li T. (2019). Noninvasive and Sensitive Optical Assessment of Brain Death. J. Biophotonics.

[10] Ferrari M., Quaresima V. (2012). A Brief Review on the History of Human Functional Near-Infrared Spectroscopy (FNIRS) Devel-opment and Fields of Application. Neuroimage.

[11] Nishizawa Y., Kanazawa T., Kawabata Y., Matsubara T., Maruyama S., Kawano M., Kinoshita S., Koh J., Matsuo K., Yoneda H. (2019). FNIRS Assessment during an Emotional Stroop Task among Patients with Depression: Replication and Extension. Psychiatry Investig..

[12] Koike S., Satomura Y., Kawasaki S., Nishimura Y., Kinoshita A., Sakurada H., Yamagishi M., Ichikawa E., Matsuoka J., Okada N. (2017). Application of Functional near Infrared Spectroscopy as Supplementary Examination for Diagnosis of Clinical Stages of Psychosis Spectrum. Psychiatry Clin. Neurosci..

[13] Devezas M.Â.M. (2021). Shedding Light on Neuroscience: Two Decades of Functional Near-infrared Spectroscopy Applications and Advances from a Bibliometric Perspective. J. Neuroimaging.

[14] Ho C.S., Chan Y.L., Tan T.W., Tay G.W., Tang T.B. (2022). Improving the Diagnostic Accuracy for Major Depressive Disorder Using Machine Learning Algorithms Integrating Clinical and Near-Infrared Spectroscopy Data. J. Psychiatr. Res..

[15] Sayar-Akaslan D., Baskak B., Kir Y., Kusman A., Yalcinkaya B., Çakmak I.B., Munir K. (2021). Cortical Activity Measured by Functional near Infrared Spectroscopy during a Theory of Mind Task in Subjects with Schizophrenia, Bipolar Disorder and Healthy Controls. J. Affect. Disord..

[16] Wei Y., Chen Q., Curtin A., Tu L., Tang X., Tang Y., Xu L., Qian Z., Zhou J., Zhu C. (2021). Functional Near-Infrared Spectroscopy (FNIRS) as a Tool to Assist the Diagnosis of Major Psychiatric Disorders in a Chinese Population. Eur. Arch. Psychiatry Clin. Neurosci..

[17] Zhu H., Xu J., Li J., Peng H., Cai T., Li X., Wu S., Cao W., He S. (2017). Decreased Functional Connectivity and Disrupted Neural Network in the Prefrontal Cortex of Affective Disorders: A Resting-State FNIRS Study. J. Affect. Disord..

[18] Gao C., Zhou H., Liu J., Xiu J., Huang Q., Liang Y., Li T., Hu S. (2022). Characteristics of Frontal Activity Relevant to Cognitive Function in Bipolar Depression: An FNIRS Study. Biomed. Opt. Express.

[19] Husain S.F., Tang T.-B., Tam W.W., Tran B.X., Ho C.S., Ho R.C. (2021). Cortical Haemodynamic Response during the Verbal Fluency Task in Patients with Bipolar Disorder and Borderline Personality Disorder: A Preliminary Functional near-Infrared Spectroscopy Study. BMC Psychiatry.

[20] Ong S.K., Husain S.F., Wee H.N., Ching J., Kovalik J.-P., Cheng M.S., Schwarz H., Tang T.B., Ho C.S. (2021). Integration of the Cortical Haemodynamic Response Measured by Functional Near-Infrared Spectroscopy and Amino Acid Analysis to Aid in the Diagnosis of Major Depressive Disorder. Diagnostics.

[21] Chao J., Zheng S., Wu H., Wang D., Zhang X., Peng H., Hu B. (2021). FNIRS Evidence for Distinguishing Patients with Major Depression and Healthy Controls. IEEE Trans. Neural Syst. Rehabil. Eng..

[22] Baik S.Y., Kim J.-Y., Choi J., Baek J.Y., Park Y., Jung M., Lee S.-H. (2019). Prefrontal Asymmetry during Cognitive Tasks and Its Relationship with Suicide Ideation in Major Depressive Disorder: An FNIRS Study. Diagnostics.

[23] Kondo A., Shoji Y., Morita K., Sato M., Ishii Y., Yanagimoto H., Nakano S., Uchimura N. (2018). Characteristics of Oxygenated Hemoglobin Concentration Change during Pleasant and Unpleasant Image-recall Tasks in Patients with Depression: Compaison with Healthy Subjects. Psychiatry Clin. Neurosci..

[24] Kawano M., Kanazawa T., Kikuyama H., Tsutsumi A., Kinoshita S., Kawabata Y., Yamauchi S., Uenishi H., Kawashige S., Imazu S. (2016). Correlation between Frontal Lobe Oxy-Hemoglobin and Severity of Depression Assessed Using near-Infrared Spectroscopy. J. Affect. Disord..

[25] Liu X., Sun G., Zhang X., Xu B., Shen C., Shi L., Ma X., Ren X., Feng K., Liu P. (2014). Relationship between the Prefrontal Function and the Severity of the Emotional Symptoms during a Verbal Fluency Task in Patients with Major Depressive Disorder: A Multi-Channel NIRS Study. Prog. Neuro-Psychopharmacol. Biol. Psychiatry.

[26] Akashi H., Tsujii N., Mikawa W., Adachi T., Kirime E., Shirakawa O. (2015). Prefrontal Cortex Activation Is Associated with a Discrepancy between Self- and Observer-Rated Depression Severities of Major Depressive Disorder: A Multichannel near-Infrared Spectroscopy Study. J. Affect. Disord..

[27] Husain S.F., McIntyre R.S., Tang T.-B., Abd Latif M.H., Tran B.X., Linh V.G., Thao T.P.N., Ho C.S., Ho R.C. (2021). Functional Near-Infrared Spectroscopy during the Verbal Fluency Task of English-Speaking Adults with Mood Disorders: A Preliminary Study. J. Clin. Neurosci..

[28] Feng K., Law S., Ravindran N., Chen G., Ma X., Bo X., Zhang X.-Q., Shen C., Li J., Wang Y. (2021). Differentiating between Bipolar and Unipolar Depression Using Prefrontal Activation Patterns: Promising Results from Functional near Infrared Spectroscopy (FNIRS) Findings. J. Affect. Disord..

[29] Hu S., Li X., Law S., Shen C., Yao G., Zhang X., Li J., Chen G., Xu B., Liu X. (2021). Prefrontal Cortex Alterations in Major Depressive Disorder, Generalized Anxiety Disorder and Their Comorbidity during a Verbal Fluency Task Assessed by Multi-Channel near-Infrared Spectroscopy. Psychiatry Res..

[30] Husain S.F., Tang T.-B., Yu R., Tam W.W., Tran B., Quek T.T., Hwang S.-H., Chang C.W., Ho C.S., Ho R.C. (2020). Cortical Haemodynamic Response Measured by Functional near Infrared Spectroscopy during a Verbal Fluency Task in Patients with Major Depression and Borderline Personality Disorder. EBioMedicine.

[31] Rai S., Griffiths K.R., Breukelaar I.A., Barreiros A.R., Chen W., Boyce P., Hazell P., Foster S.L., Malhi G.S., Harris A.W.F. (2021). Default-Mode and Fronto-Parietal Network Connectivity during Rest Distinguishes Asymptomatic Patients with Bipolar Disorder and Major Depressive Disorder. Transl. Psychiatry.

[32] Smitha K., Akhil Raja K., Arun K., Rajesh P., Thomas B., Kapilamoorthy T., Kesavadas C. (2017). Resting State FMRI: A Review on Methods in Resting State Connectivity Analysis and Resting State Networks. Neuroradiol. J..

[33] Feige B., Spiegelhalder K., Kiemen A., Bosch O.G., van Elst L.T., Hennig J., Seifritz E., Riemann D. (2017). Distinctive Time-Lagged Resting-State Networks Revealed by Simultaneous EEG-FMRI. Neuroimage.

[34] Khanna A., Pascual-Leone A., Michel C.M., Farzan F. (2015). Microstates in Resting-State EEG: Current Status and Future Directions. Neurosci. Biobehav. Rev..

[35] Wen D., Lang X., Zhang H., Li Q., Yin Q., Chen Y., Xu Y. (2021). Task and Non-Task Brain Activation Differences for Assessment of Depression and Anxiety by FNIRS. Front. Psychiatry.

[36] Rosenbaum D., Haipt A., Fuhr K., Haeussinger F.B., Metzger F.G., Nuerk H.-C., Fallgatter A.J., Batra A., Ehlis A.-C. (2017). Ab-errant Functional Connectivity in Depression as an Index of State and Trait Rumination. Sci. Rep..

[37] Fatt C.R.C., Jha M.K., Cooper C.M., Fonzo G., South C., Grannemann B., Carmody T., Greer T.L., Kurian B., Fava M. (2020). Effect of Intrinsic Patterns of Functional Brain Connectivity in Moderating Antidepressant Treatment Response in Major Depression. Am. J. Psychiatry.

[38] Gudayol-Ferré E., Peró-Cebollero M., González-Garrido A.A., Guàrdia-Olmos J. (2015). Changes in Brain Connectivity Related to the Treatment of Depression Measured through FMRI: A Systematic Review. Front. Hum. Neurosci..

[39] Yang H., Chen X., Chen Z.-B., Li L., Li X.-Y., Castellanos F.X., Bai T.-J., Bo Q.-J., Cao J., Chang Z.-K. (2021). Disrupted Intrinsic Functional Brain Topology in Patients with Major Depressive Disorder. Mol. Psychiatry.

[40] Williams J.B.W. (1988). A Structured Interview Guide for the Hamilton Depression Rating Scale. Arch. Gen. Psychiatry.

[41] Cope M., Delpy D.T. (1988). System for Long-Term Measurement of Cerebral Blood and Tissue Oxygenation on Newborn Infants by near Infra-Red Transillumination. Med. Biol. Eng. Comput..

[42] Strangman G., Culver J.P., Thompson J.H., Boas D.A. (2002). A Quantitative Comparison of Simultaneous BOLD FMRI and NIRS Recordings during Functional Brain Activation. Neuroimage.

[43] Mahmoudzadeh M., Dehaene-Lambertz G., Fournier M., Kongolo G., Goudjil S., Dubois J., Grebe R., Wallois F. (2013). Syllabic Discrimination in Premature Human Infants Prior to Complete Formation of Cortical Layers. Proc. Natl. Acad. Sci. USA.

[44] Huppert T.J., Diamond S.G., Franceschini M.A., Boas D.A. (2009). HomER: A Review of Time-Series Analysis Methods for near-Infrared Spectroscopy of the Brain. Appl. Opt..

[45] Molavi B., Dumont G.A. (2012). Wavelet-Based Motion Artifact Removal for Functional near-Infrared Spectroscopy. Physiol. Meas..

[46] Duan L., Zhao Z., Lin Y., Wu X., Luo Y., Xu P. (2018). Wavelet-Based Method for Removing Global Physiological Noise in Functional near-Infrared Spectroscopy. Biomed. Opt. Express.

[47] Niu H., Li Z., Liao X., Wang J., Zhao T., Shu N., Zhao X., He Y. (2013). Test-Retest Reliability of Graph Metrics in Functional Brain Networks: A Resting-State FNIRS Study. PLoS ONE.

[48] Fox M.D., Zhang D., Snyder A.Z., Raichle M.E. (2009). The Global Signal and Observed Anticorrelated Resting State Brain Networks. J. Neurophysiol..

[49] Murphy K., Birn R.M., Handwerker D.A., Jones T.B., Bandettini P.A. (2009). The Impact of Global Signal Regression on Resting State Correlations: Are Anti-Correlated Networks Introduced?. Neuroimage.

[50] Achard S., Bullmore E. (2007). Efficiency and Cost of Economical Brain Functional Networks. PLoS Comput. Biol..

[51] Stam C.J., van Straaten E.C.W. (2012). The Organization of Physiological Brain Networks. Clin. Neurophysiol..

[52] Cai L., Dong Q., Niu H. (2018). The Development of Functional Network Organization in Early Childhood and Early Adolescence: A Resting-State FNIRS Study. Dev. Cogn. Neurosci..

[53] Wang J., Wang X., Xia M., Liao X., Evans A., He Y. (2015). GRETNA: A Graph Theoretical Network Analysis Toolbox for Imaging Connectomics. Front. Hum. Neurosci..

[54] Watts D.J., Strogatz S.H. (1998). Collective Dynamics of ‘Small-World’ Networks. Nature.

[55] Latora V., Marchiori M. (2003). Economic Small-World Behavior in Weighted Networks. Eur. Phys. J. B-Condens. Matter Complex Syst..

[56] Maslov S., Sneppen K. (2002). Specificity and Stability in Topology of Protein Networks. Science.

[57] Humphries M., Gurney K., Prescott T. (2006). The Brainstem Reticular Formation Is a Small-World, Not Scale-Free, Network. Proc. R. Soc. B Biol. Sci..

[58] Uehara T., Yamasaki T., Okamoto T., Koike T., Kan S., Miyauchi S., Kira J.-I., Tobimatsu S. (2014). Efficiency of a “Small-World” Brain Network Depends on Consciousness Level: A Resting-State FMRI Study. Cereb. Cortex.

[59] Lewis J.D., Evans A.C., Pruett J.R., Botteron K., Zwaigenbaum L., Estes A., Gerig G., Collins L., Kostopoulos P., McKinstry R. (2014). Network Inefficiencies in Autism Spectrum Disorder at 24 Months. Transl. Psychiatry.

[60] Liu J., Li M., Pan Y., Lan W., Zheng R., Wu F.-X., Wang J. (2017). Complex Brain Network Analysis and Its Applications to Brain Disorders: A Survey. Complexity.

[61] van den Heuvel M.P., Sporns O. (2013). Network Hubs in the Human Brain. Trends Cogn. Sci..

[62] Xia M., Wang J., He Y. (2013). BrainNet Viewer: A Network Visualization Tool for Human Brain Connectomics. PLoS ONE.

[63] Benjamini Y., Hochberg Y. (1995). Controlling the False Discovery Rate: A Practical and Powerful Approach to Multiple Testing. J. R. Stat. Soc. Ser. B.

[64] Hou Y., Song B., Hu Y., Pan Y., Hu Y. (2020). The Averaged Inter-Brain Coherence between the Audience and a Violinist Predicts the Popularity of Violin Performance. Neuroimage.

[65] Pan Y., Dikker S., Zhu Y., Yang C., Hu Y., Goldstein P. (2022). Instructor-Learner Body Coupling Reflects Instruction and Learning. NPJ Sci. Learn..

[66] Kosinski M., Stillwell D., Graepel T. (2013). Private Traits and Attributes Are Predictable from Digital Records of Human Behavior. Proc. Natl. Acad. Sci. USA.

[67] Chang C.-C., Lin C.-J. (2011). LIBSVM: A Library for Support Vector Machines. ACM Trans. Intell. Syst. Technol..

[68] Tzourio-Mazoyer N., Landeau B., Papathanassiou D., Crivello F., Etard O., Delcroix N., Mazoyer B., Joliot M. (2002). Automated Anatomical Labeling of Activations in SPM Using a Macroscopic Anatomical Parcellation of the MNI MRI Single-Subject Brain. Neuroimage.

[69] Ding Y.-D., Chen X., Chen Z.-B., Li L., Li X.-Y., Castellanos F.X., Bai T.-J., Bo Q.-J., Cao J., Chang Z.-K. (2022). Reduced Nucleus Accumbens Functional Connectivity in Reward Network and Default Mode Network in Patients with Recurrent Major Depressive Disorder. Transl. Psychiatry.

[70] Yan C.-G., Chen X., Li L., Castellanos F.X., Bai T.-J., Bo Q.-J., Cao J., Chen G.-M., Chen N.-X., Chen W. (2019). Reduced Default Mode Network Functional Connectivity in Patients with Recurrent Major Depressive Disorder. Proc. Natl. Acad. Sci. USA.

[71] Suo X., Lei D., Li L., Li W., Dai J., Wang S., He M., Zhu H., Kemp G.J., Gong Q. (2018). Psychoradiological Patterns of Small-World Properties and a Systematic Review of Connectome Studies of Patients with 6 Major Psychiatric Disorders. J. Psychiatry Neurosci..

[72] Sinha P., Reddy R.V., Srivastava P., Mehta U.M., Bharath R.D. (2019). Network Neurobiology of Electroconvulsive Therapy in Patients with Depression. Psychiatry Res. Neuroimaging.

[73] Repple J., Gruber M., Mauritz M., de Lange S.C., Winter N.R., Opel N., Goltermann J., Meinert S., Grotegerd D., Leehr E.J. (2022). Shared and Specific Patterns of Structural Brain Connectivity Across Affective and Psychotic Disorders. Biol. Psychiatry.

[74] Zhang R., Kranz G.S., Zou W., Deng Y., Huang X., Lin K., Lee T.M.C. (2020). Rumination Network Dysfunction in Major De-pression: A Brain Connectome Study. Prog. Neuro-Psychopharmacol. Biol. Psychiatry.

[75] Park C., Wang S.-M., Lee H.-K., Kweon Y.-S., Lee C.T., Kim K.-T., Kim Y.-J., Lee K.-U. (2014). Affective State-Dependent Changes in the Brain Functional Network in Major Depressive Disorder. Soc. Cogn. Affect. Neurosci..

[76] Yu Z., Qin J., Xiong X., Xu F., Wang J., Hou F., Yang A. (2020). Abnormal Topology of Brain Functional Networks in Unipolar Depression and Bipolar Disorder Using Optimal Graph Thresholding. Prog. Neuro-Psychopharmacol. Biol. Psychiatry.

[77] Wang S., Gong G., Zhong S., Duan J., Yin Z., Chang M., Wei S., Jiang X., Zhou Y., Tang Y. (2020). Neurobiological Com-monalities and Distinctions among 3 Major Psychiatric Disorders: A Graph Theoretical Analysis of the Structural Connectome. J. Psychiatry Neurosci..

[78] Hasanzadeh F., Mohebbi M., Rostami R. (2020). Graph Theory Analysis of Directed Functional Brain Networks in Major Depressive Disorder Based on EEG Signal. J. Neural Eng..

[79] Long Z., Duan X., Wang Y., Liu F., Zeng L., Zhao J., Chen H. (2015). Disrupted Structural Connectivity Network in Treat-ment-Naive Depression. Prog. Neuro-Psychopharmacol. Biol. Psychiatry.

[80] Ye M., Qing P., Zhang K., Liu G. (2016). Altered Network Efficiency in Major Depressive Disorder. BMC Psychiatry.

[81] Xu X., Tang R., Zhang L., Cao Z. (2019). Altered Topology of the Structural Brain Network in Patients with Post-Stroke Depression. Front. Neurosci..

[82] Meng C., Brandl F., Tahmasian M., Shao J., Manoliu A., Scherr M., Schwerthöffer D., Bäuml J., Förstl H., Zimmer C. (2014). Aberrant Topology of Striatum’s Connectivity Is Associated with the Number of Episodes in Depression. Brain.

[83] Yan M., He Y., Cui X., Liu F., Li H., Huang R., Tang Y., Chen J., Zhao J., Xie G. (2021). Disrupted Regional Homogeneity in Melancholic and Non-Melancholic Major Depressive Disorder at Rest. Front. Psychiatry.

[84] Zhang H., Qiu M., Ding L., Mellor D., Li G., Shen T., Peng D. (2019). Intrinsic Gray-Matter Connectivity of the Brain in Major Depressive Disorder. J. Affect. Disord..

[85] Lee J.S., Kang W., Kang Y., Kim A., Han K.-M., Tae W.-S., Ham B.-J. (2021). Alterations in the Occipital Cortex of Drug-Naïve Adults with Major Depressive Disorder: A Surface-Based Analysis of Surface Area and Cortical Thickness. Psychiatry Investig..

[86] Zhang Y.-N., Li H., Shen Z.-W., Xu C., Huang Y.-J., Wu R.-H. (2021). Healthy Individuals vs Patients with Bipolar or Unipolar Depression in Gray Matter Volume. World J. Clin. Cases.

[87] Dong Q., Liu J., Zeng L., Fan Y., Lu X., Sun J., Zhang L., Wang M., Guo H., Zhao F. (2020). State-Independent Micro-structural White Matter Abnormalities in Major Depressive Disorder. Front. Psychiatry.

[88] Wang Y., Yang Z. (2022). Aberrant Pattern of Cerebral Blood Flow in Patients with Major Depressive Disorder: A Meta-Analysis of Arterial Spin Labelling Studies. Psychiatry Res. Neuroimaging.

[89] Cheng C., Dong D., Jiang Y., Ming Q., Zhong X., Sun X., Xiong G., Gao Y., Yao S. (2019). State-Related Alterations of Spontaneous Neural Activity in Current and Remitted Depression Revealed by Resting-State FMRI. Front. Psychol..

[90] Ma X., Liu J., Liu T., Ma L., Wang W., Shi S., Wang Y., Gong Q., Wang M. (2019). Altered Resting-State Functional Activity in Medication-Naive Patients with First-Episode Major Depression Disorder vs. Healthy Control: A Quantitative Meta-Analysis. Front. Behav. Neurosci..

[91] Foland-Ross L.C., Gotlib I.H. (2012). Cognitive and Neural Aspects of Information Processing in Major Depressive Disorder: An In-tegrative Perspective. Front. Psychol..

[92] Teng C., Zhou J., Ma H., Tan Y., Wu X., Guan C., Qiao H., Li J., Zhong Y., Wang C. (2018). Abnormal Resting State Activity of Left Middle Occipital Gyrus and Its Functional Connectivity in Female Patients with Major Depressive Disorder. BMC Psy-chiatry.

[93] Whitfield-Gabrieli S., Ford J.M. (2012). Default Mode Network Activity and Connectivity in Psychopathology. Annu. Rev. Clin. Psychol..

